# Comparative Study of 5-Aminolevulinic Acid-Mediated Photodynamic Therapy and the Loop Electrosurgical Excision Procedure for the Treatment of Cervical High-Grade Squamous Intraepithelial Lesions

**DOI:** 10.3390/pharmaceutics16050686

**Published:** 2024-05-20

**Authors:** Xiaoyun Wang, Xiaoming Xu, Yaxi Ma, Yixin Tang, Zheng Huang

**Affiliations:** 1 Department of Gynecology, The Second Affiliated Hospital of Zhejiang University School of Medicine, Hangzhou 310009, China; 2 Department of Pathology, The Second Affiliated Hospital of Zhejiang University School of Medicine, Hangzhou 310009, China; 3 MOE Key Laboratory of Medical Optoelectronics Science and Technology, Fujian Normal University, Fuzhou 350117, China

**Keywords:** HSIL, ALA-PDT, LEEP, efficacy, HPV

## Abstract

The loop electrosurgical excision procedure (LEEP) is a common treatment for cervical intraepithelial neoplasia (CIN). Photodynamic therapy (PDT) mediated by 5-aminolevulinic acid (ALA) is a non-invasive modality that has been used for treating precancerous diseases and HPV infections. This comparative study evaluated the efficacy and safety of ALA PDT and the LEEP in the treatment of cervical high-grade squamous intraepithelial lesions (HSILs). Patient records were reviewed and HSIL patients with HPV infections (24–51 years old) who underwent PDT or LEEP treatment were selected. Efficacy was analyzed blindly based on HPV-DNA, cytology, and colposcopy-directed biopsy obtained at 6 months after treatment. Treatment-related discomfort and side effects were also analyzed. Cure rates of 88.1% and 70.0% were achieved for the PDT group and LEEP group (*p* < 0.05), respectively. HPV-negative conversion rates of 81.0% and 62.0% were achieved for the PDT group and LEEP group (*p* < 0.05), respectively. The overall lesion remission rate of the PDT group was 19% higher than that of the LEEP group. The incidence of side effects was much lower in the PDT group. These results show that ALA PDT is a feasible non-invasive treatment for cervical HSIL.

## 1. Introduction

Cervical cancer is one of the major cancer burdens in women worldwide. Globally, it was estimated there were 604,127 cervical cancer cases and 341,831 deaths in 2020 [1]. The diagnosis and treatment of cervical precancerous lesions is critical in the prevention of cervical cancer. Approximately 20–30% of cervical high-grade squamous intraepithelial lesions (HSILs) could progress to cervical cancer within 10 years; thus, early treatment of HSILs plays a pivotal role in reducing the incidence of cervical cancer [2].

A large number of studies have shown that the loop electrosurgical excision procedure (LEEP) is the most common and effective treatment for cervical precancerous lesions [3]. However, LEEP treatment can change the cervical structure and increase the abortion rate [4,5]. There is an urgent need to develop less invasive treatments for the management of cervical squamous intraepithelial lesions (SILs), particularly for women of reproductive age who strongly wish to preserve their fertility.

Photodynamic therapy (PDT) is a minimally invasive technique with high selectivity that is based on the combination of photosensitizing drug and light irradiation [6,7]. Systemic, local, and topical PDT is not only used for the treatment of several oncologic human diseases, such as skin, esophageal, head-and-neck, lung, and bladder cancers, but also for non-malignant diseases, such as age-related macular degeneration (AMD), condyloma acuminatum, actinic keratosis (AK), and actinic cheilitis (AC) [8].

In previous studies, both first- and second-generation photosensitizers have been used for PDT in cervical SILs. First-generation photosensitizers require intravenous injection, and the response rate is 90–100%, but photosensitive dermatitis (4–28%) is a more common adverse reaction because of the relatively long metabolism time [9]. Therefore, patients need to strictly avoid light for a month or longer after systemic administration of photosensitizer to avoid skin photosensitivity, which greatly limits their clinical application. On the other hand, second-generation photosensitizers, and prodrugs in particular, such as 5-aminolevulinic acid (ALA), are usually applied locally to cervical lesions, therefore reducing the side effects of cutaneous photosensitivity.

In recent years, PDT mediated by ALA has been increasingly recognized for its high cure rate of HPV-infected cervical, vaginal, and vulvar SILs [10,11,12]. Systematic reviews and meta-analyses suggest that PDT may be a practical approach in CIN (LSIL) treatment compared with placebo treatment [13]. The common ALA PDT procedure involves the topical and local application of ALA HCl to SIL tissues in the female lower-genital tract, a short period of incubation (3–4 h), and light irradiation of the endogenous photosensitizing product protoporphyrin IX (PpIX) accumulated inside lesions [14,15].

Several recent studies suggest that ALA PDT can be a safe and efficient treatment for cervical HSILs, being particularly preferred for patients who wish to preserve their fertility [16,17,18]. Interestingly, high HPV-negative conversion rates and HPV E6/E7 mRNA-negative rates are achieved after repeated ALA PDT, some accompanied by significant increases in the expression of CD4+ and CD8+ T cells and HPV E6 and E7 proteins, suggesting favorable immune responses for the long-term control of intraepithelial lesions associated with high-risk HPV (HR-HPV) infection [19,20].

Nonetheless, comparative studies are needed to further demonstrate the clinical value of ALA PDT for the management of cervical SILs. A recent retrospective study shows that for patients with cervical LSILs, the lesion and HR-HPV clearance rates after ALA PDT were close to those after the LEEP and significantly higher than those in an observation-only group [21]. A retrospective study of 4–6-month follow-ups shows that the overall effectiveness of ALA PDT in lesion and HPV regression in CIN II patients of childbearing age is similar to that of the LEEP but with fewer side effects [22]. Encouraged by these clinical observations, we conducted a retrospective study to compare the therapeutic efficacy and safety of ALA PDT with those of the LEEP in HSIL patients. The 6-month follow-up showed that the overall lesion and HPV remission rates of the PDT group were higher than those of the LEEP group. The incidence of side effects was much lower in the PDT group.

## 2. Materials and Methods

### 2.1. Study Design

This was a retrospective blind study of a single hospital. Patient records were reviewed and HSIL patients with HR-HPV infections who underwent PDT or LEEP treatment during January 2021 and January 2023 were screened and those with matching ages were selected. Patient follow-up data were analyzed blindly by at least two gynecologists who were not involved in the treatment. This study was approved by the ethics committee of our institution prior to patient enrollment and written informed consent was obtained from all patients.

### 2.2. Study Population

The patients had HSILs confirmed by colposcopy cervical biopsy and HR-HPV infections with or without abnormal cytology. The patients were counseled about their treatment options and those that received PDT or LEEP treatment were screened.

The inclusion criteria were as follows: (1) no surgery for a cervical disease in the past year, (2) confirmed HSIL by colposcopy cervical biopsy and HR-HPV infection, (3) satisfactory colposcopy examination including visibility of the entire transformation zone (TZ) and the entire lesion margin.

The exclusion criteria were as follows: (1) unsatisfactory colposcopy evaluation, (2) lesion in the cervical canal, (3) received physical therapy and surgery for a cervical disease in the past year, (4) received adjuvant therapy or other treatment after PDT or LEEP, (5) serious comorbidity requiring concomitant treatment (e.g., heart disease, arrhythmia, or active infection), (6) pregnancy, and (7) hypersensitivity to porphyrin or ALA.

### 2.3. Diagnosis

HPV DNA typing was performed using gene probes (Jiangsu Jianyou Medical Technology Co., Ltd., Danyang, China) that detect 24 types of HPV (low-risk HPV 6, 11, 42, 43, 44, 81, and 83; HR-HPV 16, 18, 31, 33, 35, 39, 45, 51, 52, 53, 56, 58, 59, 66, 68, 73, and 82). Cervical exfoliated cell collection and cytology assays were performed using a Thinprep cytologic test (TCT) kit (Hologic, Inc., Marlborough, MA, USA). The colposcopy-directed biopsy and histologic evaluation were also performed according to the 2020 World Health Organization Classification [23].

### 2.4. Treatment Procedures

#### 2.4.1. ALA PDT

The vagina and cervix were cleaned with sterile 5% sodium chloride prior to the application of ALA. The ALA HCl solution (20%) was freshly prepared by mixing ALA HCl powder (Shanghai Fudan Zhangjiang Biopharmaceutical Co. Ltd., Shanghai, China) with temperature-sensitive gel. The amount of ALA administered was determined by the cervical lesion size plus 5 mm margin. Sterile cotton sheets of an appropriate size were soaked in the ALA mixture first and then placed over the cervical surface and cervical canal. An intravaginal plug was prepared by stuffing a condom with medical gauze and used to maintain a good contact between the ALA-soaked cotton sheets and the cervical surface. An ALA dose of 38 mg/cm^2^ and a light dose of 100 J/cm^2^ were used [14]. Light irradiation was carried out 4 h after ALA incubation. An LED device of 630 ± 10 nm (LED-IBS, Wuhan Yage Photo-Electronic Co. Ltd., Wuhan, China) was used for the cervical surface and a cylindrical diffuser coupled with a 635 nm diode laser (LD600-C; Wuhan Yage Photo-Electronic Co. Ltd., Wuhan, China) was used for the cervical canal [15]. A total of 6 treatments were carried out at intervals of 7–14 days. Discomfort, bleeding, pain, and side effects were recorded during and after each treatment.

#### 2.4.2. LEEP

Before treatment, 5% acetic acid and iodine staining was performed to delineate the extent of the lesions. LEEPs were performed under local anesthesia (2% lidocaine injection). The selection of the loop electrode depended on the distribution of the lesions that required removal. The resection range included 2–3 mm of normal tissues around the transformation zone, in which the cervical columnar epithelium was replaced by the newly formed squamous epithelium, usually the main site of cervical cancer. All surgical excisions of tissues were performed using pathological analysis. Throughout the treatments, patients were closely observed for any discomfort, bleeding, pain, or side effects.

### 2.5. Follow-Up and Outcome Assessment

HPV-DNA typing, TCT, colposcopy, and colposcopy-directed biopsy were carried out at 6 months after the completion of treatment. Assessment was carried out by at least two gynecologists who were not involved in the treatment under the condition of not knowing the patient’s treatment choice. Each colposcopy before and after treatment was evaluated blindly by two gynecologists who have been engaged in colposcopy procedures for many years, including evaluation of the appearance of the cervix, the vinegar-whitening degree, and the iodine discoloration area. If the HPV DNA, TCT, and colposcopy cervical biopsy results were all negative, cure was defined. If the high-grade biopsy was downgraded to a low grade, remission was defined regardless of the HPV and TCT results. The pathological efficiency rates consisted of cure and remission rates. The negative rate was calculated only when all HPV types had turned negative. Patients with partial-negative HPV or HPV persistence were considered HPV-positive.

### 2.6. Statistical Analysis

All statistical analyses were performed using IBM SPSS Statistics Version 24.0. Statistical data were expressed as N (%), and the chi-square test was used for comparisons between the two groups. Differences were considered statistically significant at *p* < 0.05.

## 3. Results

### 3.1. Patient Demographic Data

A total of 92 patients of productive age met the inclusion criteria. Amongst them, 42 patients received PDT treatment (the PDT group) and 50 patients received LEEP treatment (the LEEP group). The average age of the PDT group was 29.4 years old (25–48 years old) and the average age of the LEEP group was 33.2 years old (24–51 years old). No significant differences were found in the age, HPV infection, TCT status, or lesion characteristics between the two groups (Table 1).

### 3.2. Comparison of Efficacy

Only patients whose lesions and HPV had both turned negative were considered cured. Assessment at the 6-month follow-up showed that cure rates of 88.1% and 70.0% were achieved for the PDT group and the LEEP group, respectively. The cure rate of the PDT group was significantly higher than that of the LEEP group (X^2^ = 3.95, 0.01 < *p* < 0.05).

Assessment at the 6-month follow-up also showed that there were two cases (4.8%) in the PDT group and two cases (4.0%) in the LEEP group that exhibited lesion downgrading. The overall lesion remission rate of the PDT group was approximately 19% higher than that of the LEEP group. The difference between the two groups was statistically significant (92.9% vs. 74.0%, X^2^ = 5.65, 0.01 < *p* < 0.05) (Table 2).

Although there were no lesion progression cases in either group during the 6-month follow-up assessments, there were 3 patients in the PDT group and 13 patients in the LEEP group that showed lesion persistence. In the PDT group, one patient (30 years old, HPV18/31/39/51/58/82-positive pre treatment) underwent laser therapy, one patient (31 years old, HPV 6/33/18-positive pre treatment) received LEEP treatment, and another patient (24 years old, HPV16/68/42-positive pre treatment) chose repeated PDT plus laser ablation due to the presence of thicker lesions. In the LEEP group, eight patients had an HPV16 or 18 infection and five patients had multiple HPV infections, including two with HPV16 and 18 plus another HR-HPV infection. Amongst them, five patients chose cold-knife conization, four patients continued with the PDT, three patients underwent laser therapy, and another 50-year-old patient chose total hysterectomy.

The HPV-negative conversion rate of the PDT group was approximately 20% higher than that of the LEEP group. For the PDT group, HPV negativity was obtained in 34 out of 42 patients (81.0%). Five patients (11.9%) had multiple HPV infections and showed partial-negative HPV. Three patients (7.1%) showed persistent HPV infection. For the LEEP group, 31 out of 50 patients (62.0%) obtained an HPV-negative status. Ten patients (20.0%) showed partial-negative and nine patients (18.0%) showed persistent HPV infection. Statistical analysis showed that in terms of the HPV-negative rate, there was a significant difference between the two groups (81.0% vs. 62.0%, X^2^ = 3.95, 0.01 < *p* < 0.05) (Table 3).

**Figure 1:**
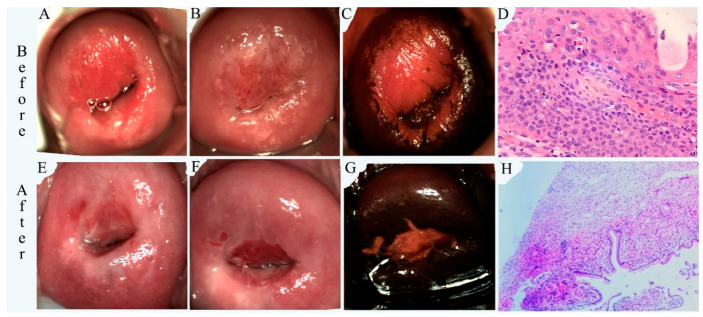
Colposcopic and histological examinations before (top panel) and after PDT (bottom panel). (**A**,**E**) Gross view, (**B**,**F**) after acetic acid test, (**C**,**G**) after iodine staining, and (**D**) H&E stain (100×) and (**H**) H&E stain (10×).

**Figure 2:**
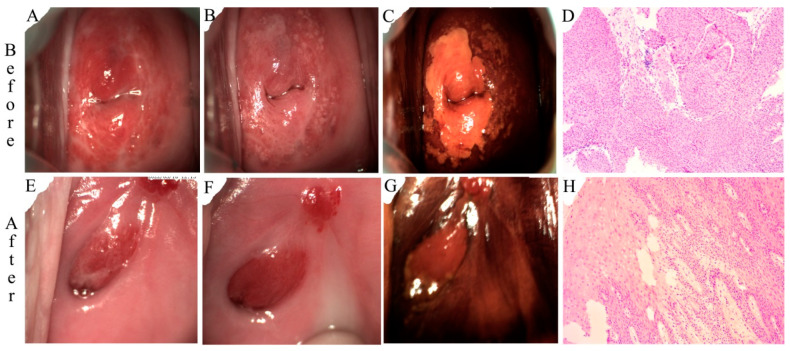
Colposcopic and histological examinations before (top panel) and after the LEEP (bottom panel). (**A**,**E**) Gross view, (**B**,**F**) after acetic acid test, (**C**,**G**) after iodine staining, and (**D**,**H**) H&E stain (10×).

Representative colposcopic and histopathological photographs obtained before and after treatment from the PDT and LEEP groups are shown in Figure 1 and Figure 2, respectively. The 24-year-old patient with HPV 39/56/58/66 infection received PDT treatment. Typical HSILs were characterized by a thick acetic acid stain and a positive iodine stain. H&E staining showed hyperplasia of the squamous epithelium accompanied by lymphocyte infiltration, indicating HSIL/CIN II. Post-treatment examination showed a normal-looking cervix and re-epithelialization of the cervical ectropion in the absence of HSILs after PDT treatment. Complete cure was achieved for this case. The 22-year-old patient with HPV 16 infection received LEEP treatment. Typical HSILs were characterized by a thick acetic acid stain and a positive iodine stain. H&E staining showed a proliferating cell compartment extended up into the whole layer. Cells were overlying koilocytosis. HSIL/CIN III was diagnosed. A normal-looking cervix and re-epithelialization of the cervical ectropion in the absence of HSILs were achieved after LEEP treatment. H&E staining showed chronic cervicitis. Although complete cure was achieved for this case, the cervical structure was altered and scar formation was visible.

### 3.3. Comparison of Side Effects

The observed side effects are summarized in Table 4. The most common side effect for both groups was increased vaginal discharge, which occurred in 83.3% of patients in the PDT group and 90% of patients in the LEEP group (*p* > 0.05). However, there were significant differences in the other side effects, including vaginal bleeding, abdominal pain, and scar formation between the two groups. Vaginal bleeding was reported in 11.9% (5/42) of patients in the PDT group, which was minimal (1–2 mL). Abdominal pain was observed in 40.5% (17/42) of patients in the PDT group, while 35.7% of the patients complained of mild pain (VAS scores: 1–4) and 4.8% complained of moderate pain (VAS scores 5–7). These side reactions were tolerable and were alleviated 3–7 days post treatment without requiring intervention. No patients experienced cutaneous photosensitization and no patients had cervical scarring after PDT treatment.

All patients had vaginal bleeding after the LEEP and 96% (48/50) of patients suffered from heavy bleeding (>2 mL). Among them, five patients came to the emergency department 10–14 days after surgery due to secondary hemorrhage and were treated with compression using vaginal gauze for 24 h. Despite local anesthesia, all patients experienced abdominal pain during or after the LEEP, which included 40% having mild pain (VAS scores 1–4) and 60% having moderate pain (VAS scores 5–7). No patients had severe pain. In addition, 20% of patients showed cervical scarring after the LEEP. A 35-year-old nulliparous woman developed cervical canal adhesion and received stenosis 3 months after surgery, followed by reoperation.

## 4. Discussion

Conventional methods for the treatment of cervical HSILs, such as excision and ablation, are invasive and may cause hemorrhages, infection, cervical deformation, cervical canal stenosis, spontaneous abortion, pre-term birth, and even sexual dysfunction [24,25]. The LEEP has become the leading treatment for HSILs, but also cause similar complications [3,4,26]. The dilemma in treating cervical HSILs is how to achieve complete excision of the lesion to minimize the risk of cervical cancer while concurrently reducing complications and preserving the integrity of the cervix. Clinically, more and more HSIL patients have fertility preservation needs that cause them to choose treatments that are less invasive [27].

ALA is an important precursor of heme biosynthesis. Exogenous supply of ALA to precancerous and cancerous lesions can lead to endogenous generation of potent photosensitizer PpIX. Its fluorescent and photodynamic properties offer unique theranostic tools for the examination and treatment of various precancerous and cancerous disorders [28,29]. ALA PDT has been used for cervical HPV infection, condyloma acuminatum, CIN, and other female genital disorders [13,30,31].

Our previous study of 55 cervical LSIL cases demonstrated that a pathological regression rate of about 90% and a HPV-DNA clearance rate of about 80% could be achieved for LSIL patients after PDT treatment [32]. Similar good clinical efficacy rates have been reported for PDT in the treatment of HSIL patients [11,16,17,18,19]. Currently, ALA PDT is one of options for HSIL patients in our hospital based on the available clinical data and current Chinese consensus [14]. In our practice, patients having persistent HR-HPV infection with biopsy-confirmed HSILs are counseled about their treatment options and about half would chose LEEP or PDT treatment. This retrospective comparison study intended to evaluate the efficacy and safety of ALA PDT and the LEEP in the treatment of cervical HSILs.

In order to ensure the quality of this efficacy analysis, age-matched patients were selected from each group. Both groups had similar clinical characteristics and there were no statistically significant differences between their age, HPV status, TCT status, and lesion grade (see Table 1).

It is well known that persistent HR-HPV infection increases the risk of cervical cancer [33]. HR-HPV clearance affects lesion regression in the short and long term. Previous studies show that the total HPV clearance rates at 6 months after PDT are 64.6–86.36% [17,19,34,35,36]. The HPV clearance rate found in this study is within this range. After the 6-month follow-up period, the HPV-negative rate in the PDT group was 81.0% (see Table 3). HPV tests were still positive in the remaining eight patients, and five of them with multiple HPV infections partially turned negative. In general, the HPV clearance rate might be lower than the lesion clearance rate obtained after treatment. Although the HPV infection might continue to turn negative over time, it should be noted that a persistent HPV infection could lead to the re-development of the cervical lesion. Therefore, long-term follow-up should be encouraged for patients with a persistent HPV infection. It is also critical to treat the cervical canal at the same time to ensure the thorough clearance of the endocervical SIL and HPV infection, which would only be guaranteed with the administration of sufficient ALA and light doses [14,37].

A previous study reported that 88 out of 99 (77.78%) patients with HSILs showed lesion regression at a 6-month follow-up after ALA PDT [18]. A single-center cohort study observed that the lesion regression rate of HSILs in a PDT-treated group was 92.0% [9]. In our study, 88.1% patients showed to be completely cured (i.e., both the HPV and HSILs disappeared) and 4.8% had their lesions reduced to LSILs after PDT treatment (see Table 2). There were no patients with progressive lesions during follow-up. The efficiency rate in the PDT group was 92.9%, which is similar to previous studies. This study demonstrates again that ALA PDT is a potentially effective method for cervical HSIL patients.

The vast majority of patients with persistent HSILs (3 in the PDT group and 13 in the LEEP group) had multiple HPV infections or HPV16/18 infections, suggesting that HSIL treatment with HPV16/18 positivity or multiple HPV infections is more challenging than that with other HPV genotypes.

The main benefits of the LEEP surgical resection include that it can be used not only for removing the lesion but also for pathological examination, thus excluding occult malignancy, confirming the diagnosis [38]. Previous reports showed cumulated clearance rates of 50% for HR-HPV infection and 70% for pathological regression (negative margins) in HSIL patients at 6 months after the LEEP [39,40]. In this study, 31 of 50 patients (62.0%) had HPV negativity and 74% patients had pathological remission after LEEP treatment, similar to the previous report. More importantly, this comparative study not only demonstrated that the lesion efficiency rate of the PDT group was significantly higher than that of the LEEP group (92.9% vs. 74.0%), but also that the HPV clearance rate of the PDT group was also significantly improved compared with that of the LEEP group (81.0% vs. 62.0%).

The following reasons might explain the high clinical efficacy of PDT: (1) the patients selected for PDT were all TZ I or II type and the lesions were visible under colposcopy, (2) ALA and light irradiation were applied to the cervical canal simultaneously, and (3) extra precaution was taken during the ALA incubation and irradiation process through repeated inspections and timely adjustments of the dressing and irradiation target.

One unique advantage of cervical PDT is its non-invasive nature, which can preserve cervical integrity and fertility function [41,42]. The main adverse reactions to PDT were local discomfort, burning sensations, and increased vaginal discharge, and only 11.9% of patients had a very small amount of bleeding and 4.8% had moderate abdominal pain (see Table 4). More importantly, there was no scar formation after PDT (see Figure 1). On the other hand, after the LEEP, 96% patients suffered from heavy bleeding, 60% suffered from moderate pain, and 20% exhibited scar formation. The cervical morphology was changed dramatically after the LEEP. Even if the patients do not have fertility needs, it will be more difficult to treat a cervical lesion if it occurs again after surgery.

In spite of its encouraging results, this study still has some limitations. Firstly, this was only a retrospective study rather than a randomized controlled trial. Secondly, the sample size used was small and the follow-up period was short. Therefore, we need multi-center and large-sample-size clinical studies to validate our results.

## 5. Conclusions

Our study demonstrated significantly higher efficiency rates and HPV clearance rates in the PDT group than in the LEEP group. Thus, ALA PDT is a safe and effective treatment option for cervical HSILs. Its non-invasive modality can maintain the structural and functional integrity of the cervix and is a feasible choice for treating cervical HSILs.

## Figures and Tables

**Table 1:** Demographic data.

	PDT (N = 42)	LEEP (N = 50)	*p*-Value
Age			>0.05
<40	37	36	
20–29	24	22	
30–39	13	14	
≥40	5	14	
Median (range)	29.4 (25–48)	33.2 (24–51)	
HPV			>0.05
16/18	22	26	
Non-16/18	20	24	
Single	23	23	
Multiple	19	27	
TCT			>0.05
NILM	15	14	
ASC-US	11	15	
LSIL	13	16	
HSIL	3	5	
Transformation zone			>0.05
TZ I	27	30	
TZ II	15	20	
Lesion characteristics			>0.05
CIN II	38	38	
CIN III	4	12	

**Table 2:** Comparison of the lesion-curing efficiency between two groups at the 6-month follow-up.

		PDT	LEEP	X^2^	*p*-Value
Effective		39 (92.9%)	37 (74.0%)	5.65	0.01 < *p* < 0.05
	Cure	37 (88.1%)	35 (70.0%)	4.39	0.01 < *p* < 0.05
	Remission	2 (4.8%)	2 (4.0%)		
Ineffective					
	Persistence	3 (7.1%)	13 (26.0%)		
	Progression	0	0		

**Table 3:** Comparison of HPV remission between two groups at the 6-month follow-up.

		PDT	LEEP	X^2^	*p*-Value
Negative		34 (81.0%)	31 (62.0%)	3.95	0.01 < *p* < 0.05
Positive					
	Partial negative	5 (11.9%)	10 (20.0%)		
	Persistence	3 (7.1%)	9 (18.0%)		

**Table 4:** Adverse reactions after treatment.

	PDT	LEEP	X^2^	*p*-Value
Bleeding	5 (11.9%)	50 (100%)	73.68	<0.01
**1–2 mL**	5 (11.9%)	2 (4%)		
**>2 mL**	0	48 (96%)		
Pain	17 (40.5%)	50 (100%)	40.87	<0.01
**Mild**	15 (35.7%)	20 (40%)		
**Moderate**	2 (4.8%)	30 (60%)		
**Severe**	0	0		
Scar formation	0	20 (20%)	21.47	<0.01
Increased vaginal discharge	35 (83.3%)	45 (90%)	0.89	>0.05

## Data Availability

Data are contained within the article.
